# Epidemiology and antimicrobial resistance of *Mycobacterium* spp. in the United Arab Emirates: a retrospective analysis of 12 years of national antimicrobial resistance surveillance data

**DOI:** 10.3389/fpubh.2024.1244353

**Published:** 2024-06-14

**Authors:** Jens Thomsen, Najiba M. Abdulrazzaq, Peter S. Nyasulu, Farida Al Hosani, Maya Habous, Stefan Weber, Fouzia Jabeen, The UAE AMR Surveillance Consortium, Abiola Senok, Godfred Antony Menezes, Carole Ayoub Moubareck, Abiola Senok, Dean B. Everett

**Affiliations:** ^1^Department of Environmental and Occupational Health, Abu Dhabi Public Health Center, Abu Dhabi, United Arab Emirates; ^2^Department of Pathology and Infectious Diseases, Khalifa University, Abu Dhabi, United Arab Emirates; ^3^Emirates Health Services Establishment, Dubai, United Arab Emirates; ^4^Department of Global Health, Faculty of Medicine and Health Sciences, Stellenbosch University, Cape Town, South Africa; ^5^Rashid Hospital, Dubai, United Arab Emirates; ^6^Sheikh Khalifa Medical City, Abu Dhabi, United Arab Emirates; ^7^Pure Labs, Abu Dhabi, United Arab Emirates; ^8^Department of Medical Microbiology and Immunology, RAK Medical and Health Sciences University, Ras Al Khaimah, United Arab Emirates; ^9^College of Natural and Health Sciences, Zayed University, Dubai, United Arab Emirates; ^10^College of Medicine, Mohammed Bin Rashid University of Medicine and Health Sciences, Dubai, United Arab Emirates; ^11^School of Dentistry, Cardiff University, Cardiff, United Kingdom; ^12^Infection Research Unit, Khalifa University, Abu Dhabi, United Arab Emirates

**Keywords:** TB, tuberculosis, *Mycobacterium tuberculosis* surveillance, antibiotics, MDR-TB, antimicrobial-resistance, UAE, MENA (Middle East and North Africa)

## Abstract

**Introduction:**

The Eastern Mediterranean Regional Office (EMRO) region accounts for almost 8% of all global *Mycobacterium tuberculosis* (TB) cases, with TB incidence rates ranging from 1 per 100,000 per year in the United Arab Emirates (UAE) to 204 per 100,000 in Djibouti. The national surveillance data from the Middle East and North Africa (MENA) region on the epidemiology and antimicrobial resistance trends of TB, including MDR-TB remains scarce.

**Methods:**

A retrospective 12-year analysis of *N* = 8,086 non-duplicate diagnostic *Mycobacterium tuberculosis* complex (MTB complex) isolates from the UAE was conducted. Data were generated through routine patient care during the 2010–2021 years, collected by trained personnel and reported by participating surveillance sites to the UAE National Antimicrobial Resistance (AMR) Surveillance program. Data analysis was conducted with WHONET, a windows-based microbiology laboratory database management software developed by the World Health Organization Collaborating Center for Surveillance of Antimicrobial Resistance, Boston, United States (https://whonet.org/).

**Results:**

A total of 8,086 MTB-complex isolates were analyzed. MTB-complex was primarily isolated from respiratory samples (sputum 80.1%, broncho-alveolar lavage 4.6%, pleural fluid 4.1%). Inpatients accounted for 63.2%, including 1.3% from ICU. Nationality was known for 84.3% of patients, including 3.8% Emiratis. Of UAE non-nationals, 80.5% were from 110 countries, most of which were Asian countries. India accounted for 20.8%, Pakistan 13.6%, Philippines 12.7%, and Bangladesh 7.8%. Rifampicin-resistant MTB-complex isolates (RR-TB) were found in 2.8% of the isolates, resistance to isoniazid, streptomycin, pyrazinamide, and ethambutol, was 8.9, 6.9, 3.4 and 0.4%, respectively. A slightly increasing trend of resistance among MTB-complex was observed for rifampicin from 2.5% (2010) to 2.8% (2021).

**Conclusion:**

Infections due to MTB-complex are relatively uncommon in the United Arab Emirates compared to other countries in the MENA region. Most TB patients in the UAE are of Asian origin, mainly from countries with a high prevalence of TB. Resistance to first line anti-tuberculous drugs is generally low, however increasing trends for MDR-TB mainly rifampicin linked resistance is a major concern. MDR-TB was not associated with a higher mortality, admission to ICU, or increased length of hospitalization as compared to non-MDR-TB.

## 1 Introduction

Tuberculosis (TB) is one of the leading causes of death worldwide (1). It is estimated that one quarter of the global population has been infected with *Mycobacterium tuberculosis* (MTB) bacilli (2), but the majority of those infected do not develop TB (3–5). Of those who do develop TB each year, about 75% are adults, with more cases among men than women. TB typically affects the lungs (pulmonary TB) but can also spread to affect other sites (extra pulmonary TB) (1). Without treatment, TB is incurable and results in a high TB associated mortality rate (1, 6, 7). With existing treatments (a 4–6 months course of anti-TB drugs) around 85% of those infected can be cured (1). Current estimates suggest that up to a quarter of the world’s population are carriers of latent TB infection (LTBI) (8).

During the COVID-19 pandemic, TB care services were disrupted, which led to an increase in the number of TB cases and TB related deaths worldwide at the time (1). As such, the COVID-19 pandemic contributed to reversing a decade of global TB control progress (9–12). For the first time since 2012, TB death rates began to increase, but in 2022 this trend was reversed (1). However, time is running out to deliver on the 2018 UN General Assembly commitment to decrease TB morbidity and mortality (13). The Global Plan to End TB (2023–2030), aims to end TB as a public health challenge by 2030; this is the same projected year governments globally have committed to achieving the United Nations (UN) Sustainable Development Goals (SDG) (14). SDG target 3.3 commits to ending the epidemics of AIDS, tuberculosis, malaria and neglected tropical diseases and combat hepatitis, waterborne diseases and other communicable diseases by 2030 (15).

However, the emergence of MDR-TB remains a significant threat to this aim. MDR-TB is defined as combined resistance to isoniazid and rifampicin, the two most effective first-line TB treatments. More than 500,000 people are newly diagnosed annually with multidrug- or rifampicin-resistant TB (MDR/RR-TB) (1). The emergence of MTB drug resistance has been driven by the inappropriate use of anti-TB medicine, through incorrect prescription by health care providers, poor quality drugs, or poor adherence to treatment among TB patients or defaulting treatment prematurely (16). The burden of drug-resistant TB has increased by 3% between 2020 and 2021, where 450,000 incident cases of rifampicin-resistant tuberculosis were reported (1). Russia and other countries in eastern Europe and central Asia reported the highest proportions (>50%) of MDR or rifampicin-resistant TB among the previously treated individuals (1). MDR-TB has become one of the major public health crises in the control of TB globally.

With the development of extensive drug resistant TB (pre-XDR-TB, XDR-TB) treatment efforts to counter resistance have become more and more challenging. Pre-XDR-TB is defined as TB caused by MTB-complex strains that fulfill the definition of multidrug resistant and rifampicin-resistant TB (MDR/RR-TB) and which are also resistant to any fluoroquinolone (17). XDR-TB is TB that is resistant to rifampicin, plus any fluoroquinolone, plus at least one of either bedaquiline or linezolid (1). Pre-XDR-TB and XDR-TB leave the affected patients with very limited treatment options and lead to poor treatment outcome, i.e., prolonged hospitalization and or death (17, 18). The emergence and spread of MDR-, pre-XDR- and XDR-TB continue to impede global efforts to curb the disease.

The most effective public health approach to controlling infections is through vaccination. Bacillus Calmette-Guérin (BCG) vaccine remains the only licensed vaccine against TB used for global control. It also remains the most widely used of all vaccines globally, with approximately 100 million children receiving it each year (19). However, the vaccine only protects children against TB and suffers from declining efficacy year on year (20). Furthermore, it does not provide protection against adult pulmonary TB (21) and lacks any additive benefit following revaccination of healthy and active TB cases (21). To circumvent these limitations there are now extensive efforts and investment into TB vaccine development with the goal of improving effectiveness. It is hoped that more effective TB vaccines for preventive and therapeutic applications in humans may become a reality in the near future.

The WHO Eastern Mediterranean Regional Office (EMRO) region accounts for almost 8% of the global burden of TB including MDR/RR-TB burden. In this region the TB incidence rates ranges from 1 per 100,000 per year to 204 per 100,000 in the United Arab Emirates and Djibouti, respectively (22). The national surveillance data from the EMRO/ Middle East and North Africa (MENA) region on the epidemiology of TB and antimicrobial resistance trends, including MDR-TB though remains scarce (23). In Kuwait almost 800 culture-confirmed TB cases are detected every year translating into 24 cases per 100,000 population with an incidence rate of 1.1% for MDR-TB (24, 25). Expatriates comprise almost 70% of Kuwait’s total population of the approximate 4.7 million individuals living in the country. Until recently, rifampicin-resistant TB/MDR-TB and XDR-TB among Kuwaiti subjects were infrequent (25, 26). In Saudi Arabia, TB incidence has remained static but resistance is increasing (27). The incidence rate ranges from 6 to 14 cases per 100,000 population (28–30). In 2019, the total number of new cases of pulmonary TB was reported in one study as 2,264 with an overall incidence rate of 6.6 per 100,000 population (31). Oman has a population of 4.6 million, of which 41% are expatriates (32). TB data is sparse from Oman (33–36). The rates of all forms of TB notification have steadily decreased to <100 cases per million since 1991 (37). One study conducted in 2022 found that from 501,290 visa applicants screened, 436 (0.09%) had X-ray findings suggestive of TB. Among the 436, TB was confirmed in 53, giving an overall prevalence of 10.6 per 100,000 applicants (37).

### 1.1 United Arab Emirates

Studies from across the United Arab Emirates (UAE) have reported various rates of resistance across first- and second-line TB treatments over time. One study from 2004 to 2008 in Sharjah Emirate reported streptomycin resistance at 14%, and MDR-TB at 5% (38). A 2013 study reported a pulmonary TB prevalence in the visa screening program of 38 per 100,000. Cases in the program were predominantly within low-income workers such as nursery workers, house helpers and private drivers (39). Other studies in the UAE have reported that 8–10% of Emirati medical students and 0.5% of Emirati children have LTBI following a TB interferon gamma release assay positive result (IGRA) (40, 41). A recent study published in 2020 found that among 1,116 newly identified TB patients within the Dubai Health Authority during 2016–2019, 6.9% of cases had MDR TB (42). In the same study, resistance to at least one or more first line anti-TB medicine was 17.3% which was higher than 6% in Oman but lower than 32.4% in Jordan (43). The study also confirmed that isoniazid resistance was more prevalent in Dubai (42). To build on previous UAE studies, we report for the first time MTB-complex AMR trends across the UAE over a 12-year period (2010–2021) collected through a National Surveillance Program.

## 2 Methods

### 2.1 Study design and data sources

A multi-institutional retrospective observational study was conducted between 2010 and 2021 in the UAE across all Emirates. The study used demographic and microbiological data collected by trained personnel as part of the UAE national AMR surveillance program. The data is collected and analyzed through a unified WHONET platform.^1^ The data presented here comprises of all nationals, including Emiratis and residents across the UAE.

### 2.2 The UAE national AMR surveillance program

The UAE national AMR surveillance program was initiated in 2010 in the Abu Dhabi Emirate with 6 hospitals and 16 Healthcare Centers/Clinics participating. Additional sites were recruited over the years, starting with only 22 participating sites in 2010, which is the first year during which the study started, and were located only in the Emirate of Abu Dhabi. By 2021, the program includes a total of 317 surveillance sites from all the 7 Emirates includes 84 hospitals and 233 healthcare centers/clinics, representing all the seven emirates of the country (44).

### 2.3 Enrollment of national AMR surveillance sites

In 2010 the Department of Health Abu Dhabi (DoH) established AMR surveillance in the Emirate of Abu Dhabi and started enrolling healthcare facilities. Based on this experience, in 2014, the Ministry of Health and Prevention (MOHAP) established AMR surveillance at the national level. UAE healthcare facilities enrolled were from one Emirate only (Abu Dhabi) during the initial years (2010 to 2012), from five Emirates (2013), and since then, from all seven Emirates (2014 to 2021).

Clinical and antimicrobial susceptibility testing data on *M. tuberculosis* isolates is obtained from three laboratories (two in Abu Dhabi; one in Dubai), which were receiving MTB-complex samples from all seven Emirates. The laboratories were Union71/PureLab at Sheikh Khalifa Medical City (Abu Dhabi) and the National Reference Laboratory (NRL) in Abu Dhabi; and Rashid Hospital TB laboratory in Dubai. These three laboratories represent the vast majority of TB-complex isolates in the UAE, while very few facilities are sending their samples to reference laboratories outside of the UAE.

### 2.4 Identification of MTB complex

Identification of MTB-complex isolates was performed at the National AMR surveillance sites by medical professionals as per standard protocols and manufacturer’s instructions. Each sample was processed for smear microscopy by either Ziehl-Neelsen or auramine-O stains and conventionally cultured on solid (Lowenstein-Jensen) and liquid media (MGIT™ 960 system Mycobacteria Growth Indicator Tube, Becton Dickinson, New Jersey, United States). The MGIT™ 960 system uses a modified Middlebrook 7H9 broth base as a liquid medium (4 mL per MGIT™ tube). Samples were incubated routinely at 37°C until positivity and at least for 42 days in liquid culture and 7 weeks on solid culture. Culture-positive samples were confirmed as MTB-complex isolates by MPT64 antigen immunochromatography assay (TBcID). Samples that are *TBc Identification Test* positive are reported as MTB complex and those that test negative are tested by Cepheid GeneXpert ultra PCR. At present *M. tuberculosis* complex, *M. avium* complex and *M. intracellulare* can be detected by PCR.

### 2.5 Antimicrobial susceptibility testing of MTB-complex isolates

*In vitro* drug susceptibility testing (DST) of MTB-complex isolates was conducted using the phenotypical “critical concentration”-based method. The MGIT™ 960 system (Becton-Dickinson, New Jersey, United States) was used as per manufacturer’s instructions and MTB-complex isolates were routinely tested for susceptibility to the following five first-line antibiotics: isoniazid (0.1 μg/mL), rifampin (1.0 μg/mL), ethambutol (5.0 μg/mL), streptomycin (1.0 μg/mL), and pyrazinamide (100 μg/mL). AST is performed on the first positive culture from a patient. Where drug resistance is detected the test is repeated, where appropriate, at both standard antibiotic concentration and high-strength concentration. Where both are resistant the drug is reported as resistant. Where standard strength is resistant and high strength sensitive, the drug is reported as intermediate. At Rashid hospital Dubai TB lab only, selected MTB-complex isolates were also tested for susceptibility to second-line antibiotics (amikacin 1.0 μg/mL, capreomycin 2.5 μg/mL, kanamycin 2.5 μg/mL, moxifloxacin 0.25 μg/mL, ofloxacin 2.0 μg/mL), if resistant to first-line antibiotics.

### 2.6 Antimicrobial resistance trends in MTB complex

This was assessed by analysis of routine national level surveillance data. This data, which covers a spectrum of AMR pathogens including TB, was obtained from across the network of participating hospitals, health centers, clinics, and diagnostic laboratories in the country. These participating centers include primary, secondary, and tertiary care facilities as well as public and private entities. All data are routinely collected and analyzed using a unified platform (WHONET) and training on data collection is provided to ensure quality assurance and accuracy. The fully anonymized data include demographic data (age, gender, nationality, hospital site/location etc.), clinical and microbiological data such as specimen source and antibiogram. For the reporting of antimicrobial resistance, the interpreted and validated test result (S/I/R) as obtained from MGIT 960™ and reported by participating laboratories was used.

### 2.7 Data management and statistical analysis

Prior to analysis, data cleaning procedures were conducted to assess inconsistencies, duplicates, missing parameters and other data errors and omissions. Frequency tables were constructed to describe the characteristics of the study population. Graphic presentation of data was done to show patterns and trends of TB over the 12-year period.

Data analysis was routinely carried out using the WHONET 2023 software. For additional statistical analysis, other software packages used were IBM SPSS Statistics, version 29.0.0.0 (IBM 2022), Epi Info™ for Windows v7.2.4.0 and R-4.3.1 for Windows. Statistical significance of temporal trends for antimicrobial resistance was calculated if data from at least five consecutive years with at least 30 isolates per year was available. Statistical significance of trends is expressed as a *p*-value, calculated by a Chi-square for trend test (extended Mantel–Haenszel). For testing significant difference in mortality and ICU admission a Fishers Exact test was used while significant difference in length of stay was assessed through the weighted log-rank survival analysis. This was done to take care of differences in sample size between the comparison groups. The 95% confidence interval was determined for the proportions of resistance (%R) as well as the proportion of susceptibility (%S). This was determined based on the Wilson Score Interval with continuity correction. A *p* < 0.05 was considered statistically significant.

## 3 Results

### 3.1 Distribution of reporting sites for national AMR surveillance

**Figure 1:**
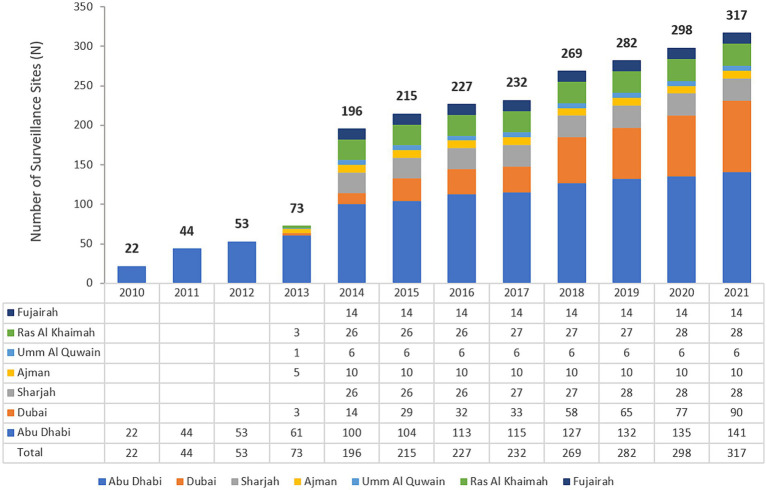
Number of national AMR surveillance sites (UAE, 2010–2021), by Year and Emirate. The bold numbers on top of the bars represent the total number of sites.

The UAE national AMR surveillance program was initiated in 2010 in the Abu Dhabi Emirate by the Department of Health Abu Dhabi (DoH) where 6 hospitals and 16 Centers/Clinics were enrolled initially. Additional sites were recruited over the years, starting with these 22 participating sites in 2010, which is the first year during which the study started, and located only in the Emirate of Abu Dhabi, to reach a total of 317 surveillance sites from the 7 Emirates, including 84 hospitals and 233 centers/clinics and representing all seven Emirates of the country in 2021. A significant increase in the number of AMR surveillance sites and labs was observed after Ministry of Health and Prevention started the national AMR surveillance program in 2014. Figure 1 presents the distribution of reporting sites for National AMR Surveillance from 2010 to 2021.

### 3.2 Bacterial population

**Figure 2:**
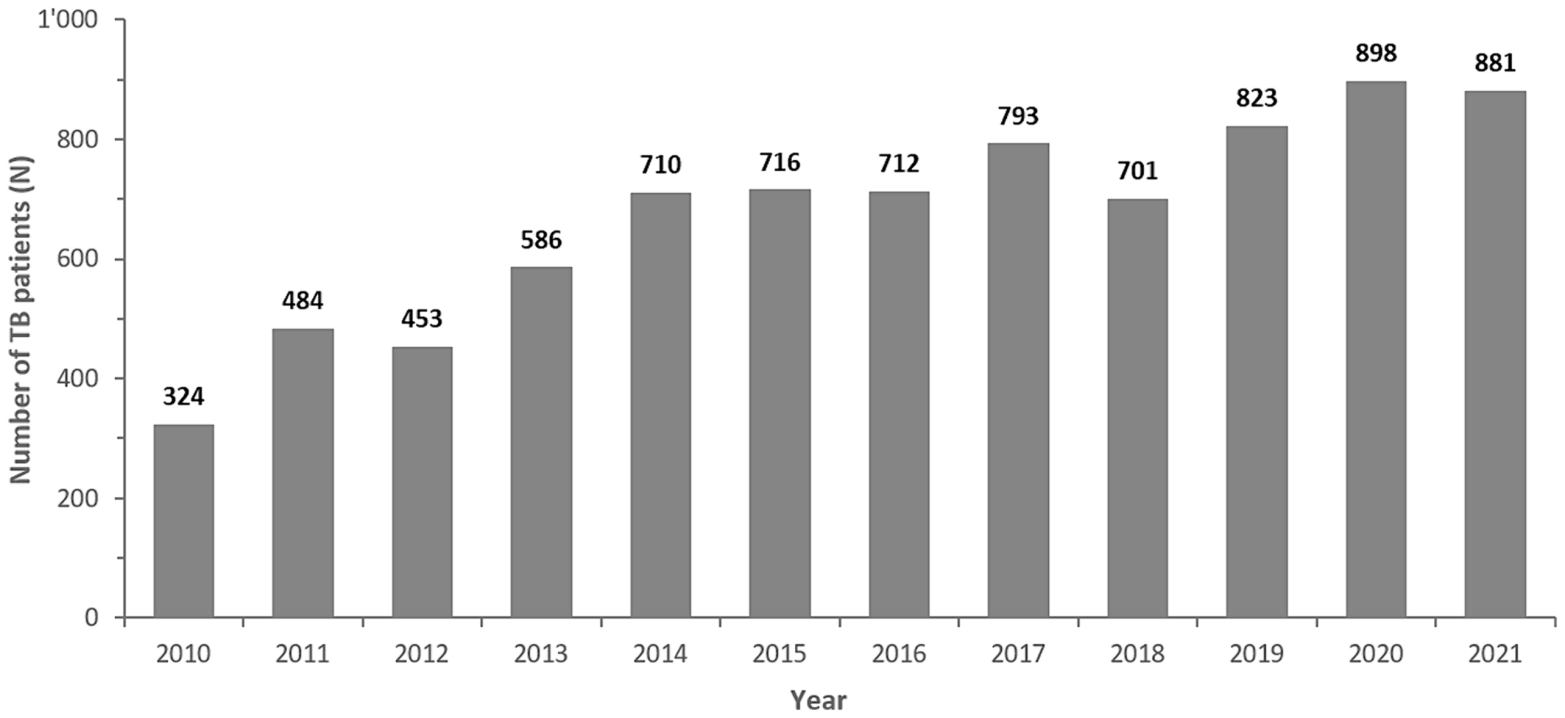
Number of reported MTB complex isolates/patients, by Year (UAE, 2010–2021). The bold numbers on top of the bars represent the number of TB-complex patients per year.

From 2010 to 2021 a total of 8,452 *Mycobacterium* spp. isolates were reported to the national AMR surveillance program. After removal of duplicate isolates and non-*M. tuberculosis*-complex isolates, a total of *n* = 8,086 *M. tuberculosis*-complex isolates remained for analysis. The numbers of MTB-complex patients had remained relatively stable from 2014 to 2018, but overall reported MTB-complex patients had risen from 324 in 2010 to 881 cases in 2021 (Figure 2).

### 3.3 Distribution of MTB patients by gender, age group, nationality status, nationality, and patient location

Demographic distribution of the patients from whom these isolates were obtained revealed a male preponderance (73.6%) with the majority of patients being in the adult age group (94.1%). Data on nationality were known for 84.3% of patients of whom 3.8% patients were Emiratis, and 80.5% patients were non-Nationals (Table 1).

**Table 1:** Distribution of MTB-complex cases by gender, age group, nationality status, and patient location (UAE, 2010–2021).

TB patients (*N* = 8,086)			
Demographic	Category	*N*	%
Gender	Male	5,952	73.6
	Female	2,126	26.3
	Unknown	8	0.0
Age group	Adult	7,611	94.1
	Newborn	284	3.5
	Pediatric	188	2.3
	Unknown	3	0.0
Nationality status	Emirati	310	3.8
	Non-Emirati	6,506	80.5
	Unknown	1,270	15.7
Patient location	ICU	109	1.3
	Inpatient	5,004	61.9
	Outpatient	2,647	32.7
	Other/unknown	326	4.0

**Figure 3:**
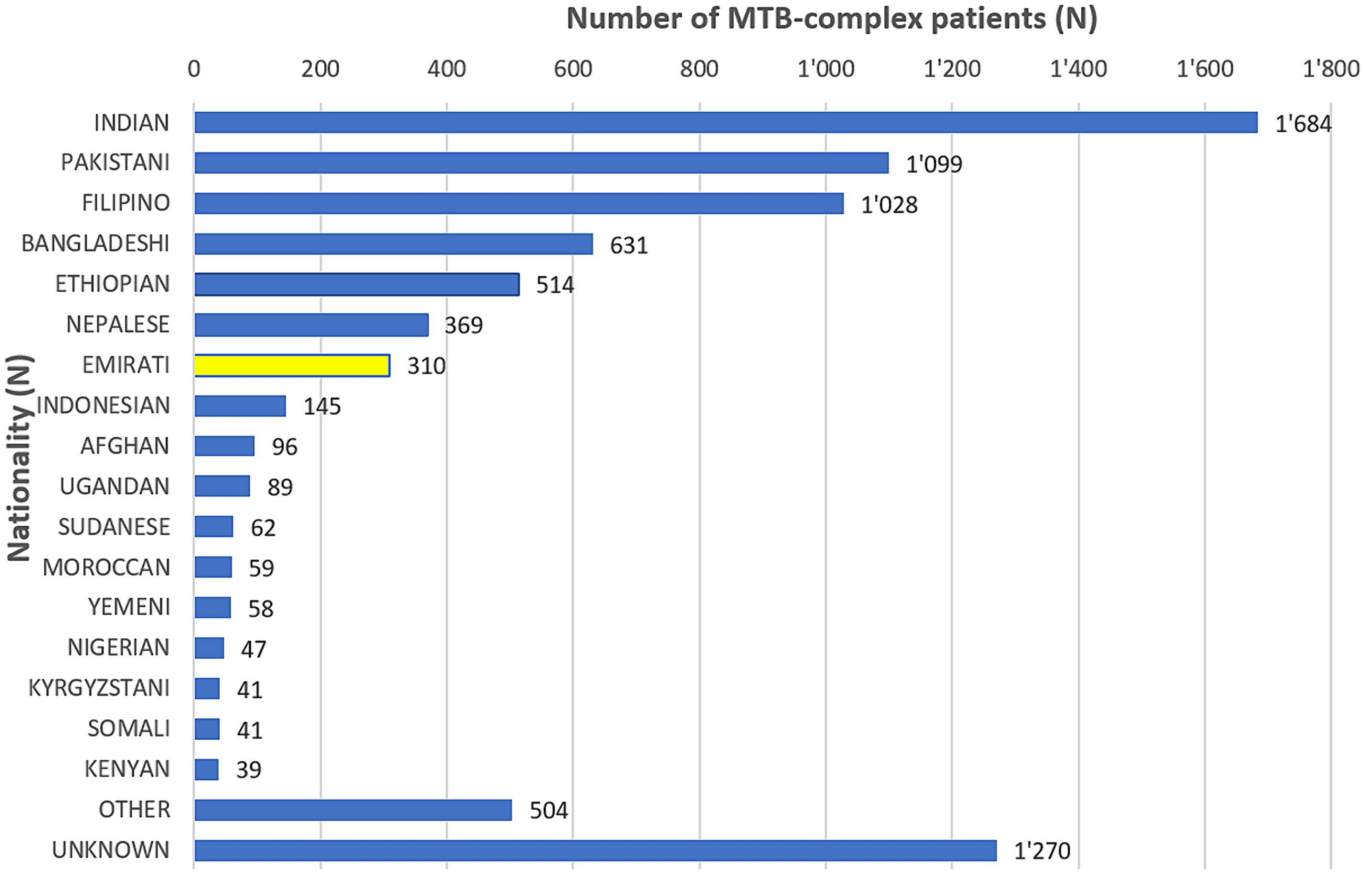
Distribution of MTB-complex patients, by Nationality (UAE, 2010–2021).

The non-Nationals were from a total of 110 countries, most commonly from Asian countries. Of these, India accounted for 20.8%, Pakistan 13.6%, Philippines 12.7%, and Bangladesh 7.8% (Figure 3). Inpatients accounted for 63.2% (5,113/8086) of which 1.3% (109/8086) were from ICU, and outpatients accounted for 32.7% (2,647/8086; Table 1).

### 3.4 Distribution of MTB-complex patients by specimen type

MTB complex was most commonly isolated from respiratory samples (90.3%, including sputum 80.1%, broncho-alveolar lavage 4.6%, pleural fluid 4.1%), whereas other sources included pus, tissue and body fluid samples from various locations (9.7%), including cerebrospinal fluid (*n* = 92, 1.1%) and blood (*n* = 17, 0.2%).

Within the UAE there are a limited numbers of TB laboratories, as most hospitals, in particular the private smaller ones, do not have Biological Safety Level 3 (BSL-3) laboratories available to screen for MTB-complex. In fact, in 2023, to the best of our knowledge, there were only three laboratories based across two Emirates (Abu Dhabi and Dubai – see methods section) that could process suspected TB samples. These three laboratories received most of the samples (estimated at >95%) from all seven Emirates in the UAE, while few facilities would send their MTB samples to laboratories abroad (estimated at <5%). The Rashid hospital TB lab, as well as the SKMC TB lab have been reporting to the national AMR surveillance program since 2010, the NRL TB lab since 2019, when it was established.

### 3.5 Antimicrobial resistance trends for MTB-complex isolates

The percentage of MTB-isolates susceptible (%S), intermediate (%I), and resistant (%R) to streptomycin, rifampin, ethambutol, isoniazid, and pyrazinamide during the study period 2010 to 2021 is shown in Table 2. The number and percentage of MTB-complex isolates non-susceptible to one, two, three, four and five first-line antibiotics is shown in Table 3.

**Table 2:** Percentage of susceptible, intermediate, and resistant MTB-complex isolates (UAE, 2010–2021).

Antibiotic	Year	Number of isolates (N)	%R	%I	%S	95% C.I. (%R)
Streptomycin	2010	319	6.6	1.3	92.2	4.2–10.0

	2011	484	6.6	2.5	90.9	4.6–9.3
	2012	451	5.1	1.6	93.3	3.3–7.7
	2013	576	5.2	1.7	93.1	3.6–7.4
	2014	712	4.6	1.7	93.7	3.3–6.5
	2015	642	4.2	3.3	92.5	2.8–6.1
	2016	319	5.3	0.0	94.7	3.2–8.6
	2017	314	6.1	0.0	93.9	3.8–9.4
	2018	308	8.4	0.0	91.6	5.7–12.3
	2019	360	8.1	0.0	91.9	5.6–11.5
	2020	485	6.0	0.0	94.0	4.1–8.6
2021	419	6.9	0.0	93.1	4.8–9.9
Rifampin	2010	320	2.5	0.0	97.5	1.2–5.1

	2011	481	2.9	0.0	97.1	1.7–5.0
	2012	451	3.5	0.0	96.5	2.1–5.8
	2013	578	3.6	0.0	96.4	2.3–5.6
	2014	712	3.2	0.0	96.8	2.1–4.9
	2015	719	2.8	0.0	97.2	1.8–4.3
	2016	712	5.3	0.0	94.7	3.9–7.3
	2017	782	3.1	0.0	96.9	2.0–4.6
	2018	704	3.7	0.0	96.3	2.5–5.4
	2019	825	4.7	0.0	95.3	3.4–6.5
	2020	894	3.0	0.0	97.0	2.0–4.4
2021	871	2.8	0.0	97.2	1.8–4.1
Ethambutol	2010	319	0.9	0.0	99.1	0.2–3.0

	2011	480	0.8	0.4	98.8	0.3–2.3
	2012	451	1.1	0.2	98.7	0.4–2.7
	2013	576	1.6	0.5	97.9	0.8–3.1
	2014	712	0.6	0.4	99.0	0.2–1.5
	2015	719	0.8	0.1	99.0	0.3–1.9
	2016	712	1.5	0.6	97.9	0.8–2.8
	2017	783	0.4	0.1	99.5	0.1–1.2
	2018	703	1.0	0.1	98.9	0.4–2.1
	2019	823	2.2	0.2	97.6	1.3–3.5
	2020	888	1.2	0.2	98.5	0.7–2.3
2021	764	0.4	0.0	99.6	0.1–1.2
Isoniazid	2010	319	12.2	0.6	87.1	8.9–16.5

	2011	484	10.5	2.3	87.2	8.0–13.7
	2012	452	7.5	2.2	90.3	5.3–10.5
	2013	578	9.0	1.6	89.4	6.9–11.7
	2014	712	8.3	0.6	91.2	6.4–10.6
	2015	719	7.6	2.6	89.7	5.9–9.9
	2016	711	9.8	2.3	87.9	7.8–12.3
	2017	782	6.5	2.2	91.3	4.9–8.5
	2018	704	9.5	1.3	89.2	7.5–12.0	
	2019	825	10.1	1.5	88.5	8.1–12.4
	2020	888	9.9	1.8	88.3	8.1–12.1
2021	766	8.9	1.2	89.9	7.0–11.2
Pyrazinamide	2010	319	11.6	0.0	88.4	8.4–15.8

	2011	462	10.4	0.0	89.6	7.8–13.6
	2012	436	10.3	0.0	89.7	7.7–13.7
	2013	572	8.2	0.0	91.8	6.2–10.9
	2014	706	6.2	0.0	93.8	4.6–8.3
	2015	715	7.0	0.0	93.0	5.3–9.2
	2016	713	8.7	0.0	91.3	6.8–11.1
	2017	785	7.9	0.0	92.1	6.2–10.1
	2018	680	10.6	0.0	89.4	8.4–13.2
	2019	824	6.1	0.0	93.9	4.6–8.0
	2020	888	3.4	0.0	96.6	2.3–4.8
2021	765	3.4	0.0	96.6	2.3–5.0

**Table 3:** Percentage of MTB-complex isolates non-susceptible to one, two, three, four, and five first-line antibiotics (UAE, 2010–2021).

Number of first line antibiotics non-susceptible	Number of TB-complex isolates (N)	Percentage of TB-complex isolates (%)
0	6,818	84.8
1	834	10.4
2	187	2.3
3	110	1.4
4	67	0.8
5	24	0.3
Total	8,040	100.0

**Figure 4:**
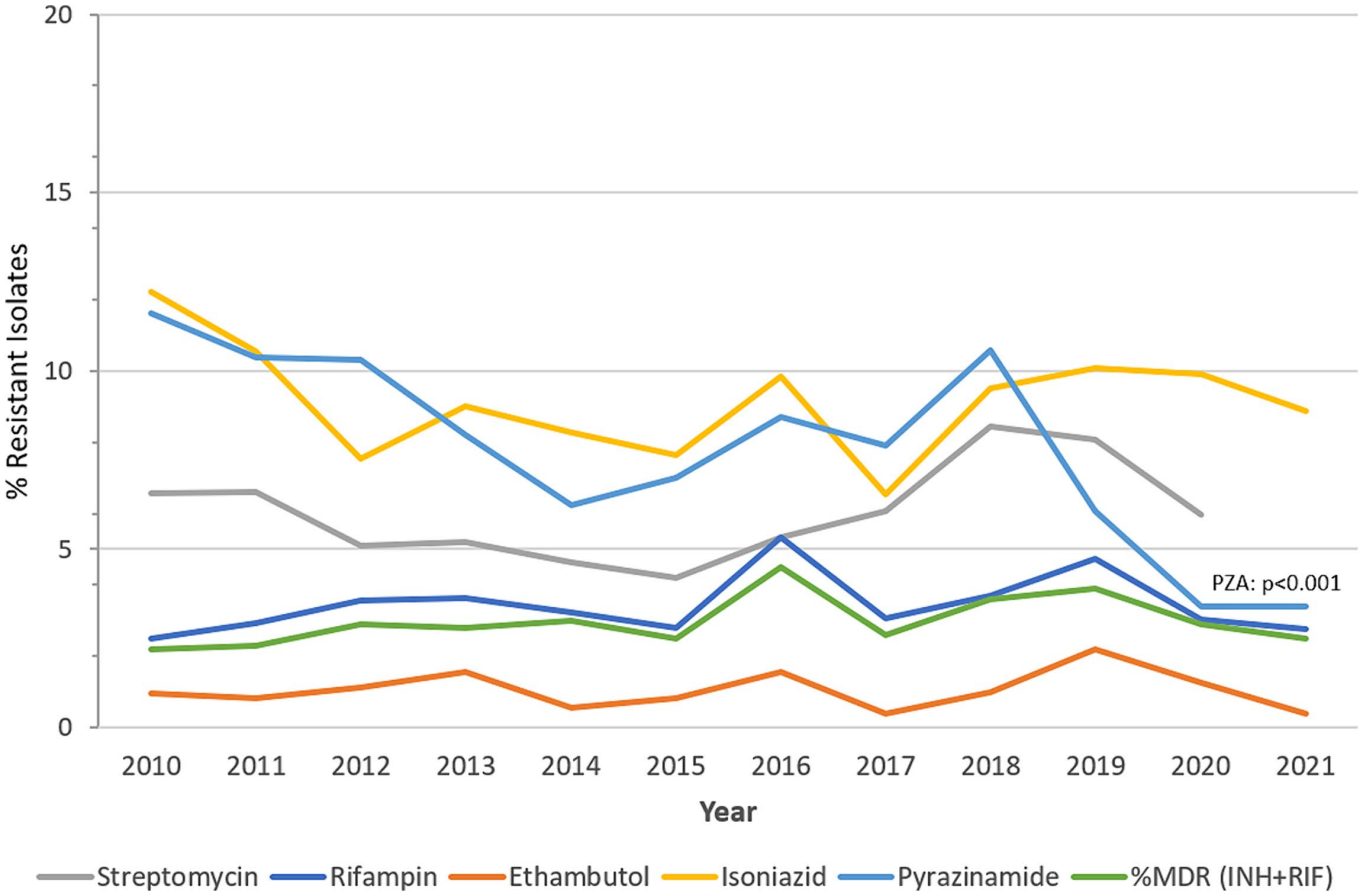
MTB-complex AMR trends over time. Trend of percentage of isolates resistant (%R) to streptomycin, rifampin, ethambutol, isoniazid, pyrazinamide, and multidrug-resistant (%MDR) (UAE, 2010-2021).

Figure 4 presents a visualization of this data, showing the annual trends of percent resistant MTB-complex isolates (%R) during the study period. During the surveillance period MTB-complex resistance levels remained relatively low across all five first-line drugs of anti-TB medicines used.

Rifampicin-resistant MTB-complex isolates (RR-TB) were found in 2.8% of the isolates (2021), resistance to isoniazid, streptomycin, pyrazinamide, and ethambutol, was 8.9, 6.9, 3.4 and 0.4%, respectively, in 2021. A slightly increasing trend of resistance among MTB-complex has been observed for rifampicin from 2.5% (2010) to 2.8% (2021), however this trend was statistically not significant (*p*-value 0.71, Chi-square 0.13747). MDR-TB, defined as resistance to both rifampicin and isoniazid showed a statistically non-significant increase from 2.2% in 2010 to 2.5% in 2021. A statistically significant decreasing trend of resistance was observed for pyrazinamide from 11.6% (2010) to 3.4% (2021; *p* < 0.001, *X*^2^ = 40.4). There was low prevalence (2%) of Pre-XDR-TB and XDR-TB in the Dubai Emirate. It is worthwhile to note that data for pre-XDR and XDR-TB among MDR-TB isolates (42) was also only available for this Emirate hence not comparable to other Emirates (Table 3).

### 3.6 Mortality rate

A subgroup analysis including the nine clinical institutions that reported mortality data was performed. In these institutions, a total of 2,036 patients developed an infection associated with non-MDR-TB of whom 19 patients died (mortality = 0.93%), while a total of 62 patients developed an infection associated with MDR-TB, of whom none was recorded to have died (mortality = 0%). Fisher’s exact test *p* = 1.00, showing no significant difference in mortality.

### 3.7 Admission to intensive care unit

A total of 7,920 patients developed an infection associated with non-MDR-TB of whom 108 patients were admitted to ICU (ICU admission rate: 1.36%) while a total of 209 patients developed an infection associated with MDR-TB, of whom 1 patient was admitted to ICU (ICU admission rate: 0.48%). There was no significant difference in ICU admission between non-MDR-TB and MDR-TB groups (Fisher’s exact test: *p* = 0.62).

### 3.8 Length of stay

A subgroup analysis including those patients for whom the date of admission as well as the date of discharge was known was performed. For those patients who developed an infection associated with non-MDR-TB (*n* = 935) the median length of stay was 15.0 days, while for those patients who developed an infection associated with MDR-TB (*n* = 20) the median length of stay was 21.5 days. However, the weighted log-rank test showed that there was no statistically significant difference in length of stay (LOS) between MDR-TB and non-MDR-TB patients (*p* = 0.21, weighted log-rank survival analysis; see also: Supplementary Figure S1).

## 4 Discussion

The global dissemination and burden of infections associated with MTB-complex is of great concern. Thus understanding the epidemiological trends of MTB-complex is critical for effective treatment and infection control strategies for the UAE to fulfill the goals of the ‘End TB’ strategy by 2030. Infections due to MTB-complex are generally low in the UAE, compared to other countries in the EMRO/MENA region (22), however the increase in labor migration and tourism poses a public health risk of spread of MTB-complex within the UAE and beyond. Nevertheless, national surveillance programs are critical for monitoring trends of MTB-complex and associated antimicrobial resistance trends and patterns over time.

This paper presents data of MTB-complex trends and associated antimicrobial resistance from a national surveillance system over a period of 12 years. Our findings provide the first comprehensive epidemiological profile of MTB-complex in the UAE and its associated AMR trends in the region, which are of course a concern for our clinical management. There has been no previously published data on TB resistance trends ever reported within the UAE until now. This data provides new insights into the epidemiological characteristics of TB cases in the UAE and demonstrates an increase in the trend of MTB-complex over the study period, but with relatively stable yearly rates since 2014, which is either a reflection of increased and better surveillance or possibly incident TB cases that would require a more detailed clinical investigation. The stable TB rates in the UAE differ with global reported trends, which saw an overall dip in reported TB cases during the COVID-19 pandemic (1). The current study utilized a significant dataset amassed over 12 years from the national AMR surveillance program. This data allowed for visualization of comprehensive trend monitoring of antibiotic resistance among the MTB-complex. The 8,086 cases of MTB-complex samples reported in this study all have laboratory-confirmed identity and detailed antibiotic resistance profiles, demonstrating the high accuracy of the data that were used in this study. The finding of a slight decline in antibiotic resistance in MTB-complex over 12 years is potentially an interesting aspect of this study (Figure 4). The samples, collected and evaluated for MTB-complex were processed at the three Emirates sites with TB laboratory infrastructure, due to the biological safety level required. However, these samples were collected from facilities across all seven Emirates of the UAE that are served by these three laboratories. These results do warrant further detailed genetic epidemiological studies moving forward. Unfortunately, at present genomic data is not part of the national surveillance dataset. Lack of genomic data is a significant limitation, particularly as there is limited data on molecular characterization of MTB-complex strains in the UAE and across the MENA region. Genomic characterization would greatly strengthen the efforts for effective identification of MTB-complex as this will help reinforce design of elimination strategies that are currently underway.

The UAE, as highlighted, is a low incidence country for TB, with an estimated rate of 1 per 100,000 (45), yet TB control efforts remains a major priority area for the UAE (46, 47). TB screening is mandatory for all expatriates applying for a work and/or residence visa in the UAE. The UAE has a cosmopolitan society with over 200 nationalities that live and work in the country. The majority of TB cases in the UAE are of Asian origin, mostly from countries with a high prevalence of TB (India, Pakistan, Bangladesh, Philippines) (1). This is not unexpected given the large expatriate and non-skilled work force population within the UAE, that has a population of 10.17 million as of 2023 (48). Indian nationals form the largest expatriate group (2.80 million or 27.49% of total UAE population), followed by Pakistani (1.29 million or 12.69%), Bangladesh (0.75 million or 7.40%) and the Philippines (0.57 million or 5. 6%). Together these total over half (53.1%) of the UAEs combined population (48). These countries also represent those with the highest burden of TB according to global estimates and are among the top 8 countries globally with the highest number of new reported TB cases in 2023 (1, 49). In addition, people from these countries accounted for almost half of all individuals with TB (3,842/8084, 47.6%) in the UAE, during the study period. Emiratis accounted for 3.8% (310/8084) of MTB-complex cases over this 12-year study period. MTB-complex in this study had a preponderance for males (5,952/8084, 73.6%) and adults (7,611/8084, 94.1%), which is in line with what would be expected from expatriate populations traveling between their home countries and the UAE. MTB-complex was more commonly found among inpatients (5,113/8084, 63.2%, including ICU, 109/8084, 1.3%). The larger proportion of the patients were enrolled in medicine (4,032/8084, 49.9%) and emergency (1765/8084, 21.8%) departments. This is encouraging as patients are actively seeking medical assistance to allow for the detection and treatment of this infection.

When looking at diagnosis, TB was overwhelmingly detected in sputum samples (6,478/8084, 80.1%), followed by pleural fluid (372/8084, 4.6%) and broncho-alveolar lavage (333/8084, 4.1%). This accounted for 88.8% of all MTB detections. Data on relatively less miliary (17/8084, 0.2%; bone marrow: 5/8084, 0.1%) and meningeal TB (92/8084, 1.1%) was also reported. This suggested that there was a lower prevalence of severe forms of TB in UAE.

MTB resistance to various drugs, whether single resistance, MDR-TB, pre-XDR-TB or XDR-TB, remain serious problems that represent great threats and challenges to human and public health (50–52). In 2021, the estimated proportion of people with TB who had MDR/RR -TB was 3.6% among new cases and 18% among those previously treated. Three countries accounted for 42% of global cases in 2021, India (26%), the Russian Federation (8.5%) and Pakistan (7.9%) (1). However, there are very few reported cases of pre-XDR-TB and XDR-TB in the UAE currently. This is positive, as the rate of MDR-TB remains low and does not appear to be increasing. In 2016 the proportion of MDR-TB was 4.5% among MTB isolates identified, which decreased to 2.5% in the 2021 calendar cycle. Resistance to first line anti-tuberculous drugs is generally low, however there is a noticeable increase in the trend of single resistance to rifampicin, peaking in 2016 at 5.3% and again in 2019 at 4.7%, but now stands at 2.5%, which is of concern and warrants close monitoring. Conversely a decreasing trend of resistance was observed for pyrazinamide, peaking in 2010 at 11.6% and is now reported to be 3.4% according to 2021 data. While the observation of lower rates of MDR-TB is positive in terms of TB control strategies, there is need to continue vigilance in effectively diagnosing and treating identified cases so that some sporadic increases in such cases is contained. Furthermore enhancing genomic surveillance would re-enforce a better understand on the evolutionary changes TB drug resistance in this population.

The mortality rate, according to our observations, was 0.9%, a sign that TB treatment adherence is optimal, ending up with favorable treatment outcomes. Similarly low admission rate ICU (0.5%), were observed among patients with MDR-TB to despite the fact that they had a longer median length of stay in hospital in general of 21.5 days, as compared to 15.0 days for patients with non-MDR-TB infections (1.4%, and 15.0 days, respectively).

## 5 Conclusion

To maintain low incidence rates and work toward achieving the End TB goals, it is critical for the UAE to continue screening the immigrant population, which includes expatriates, and other laborer migrants particularly after arrival. This should include individuals whose initial exit screening from country of origin were TB negative. Based on the data showing high incidence of TB from people who moved from high burden TB countries, there is need to reinforce screening activities on point of entry and potentially on reapplication for renewal of the work or residence visa as a strategy to curtail TB burden in the UAE.

## Data availability statement

The datasets presented in this article are not readily available because the National AMR Surveillance database managed by the UAE Ministry of Health and Prevention (MOHAP) contains confidential health information. Requests to access the datasets should be directed to https://mohap.gov.ae/.

## Ethics statement

Ethical approval for this study was provided by the Ministry of Health and Prevention Research Ethics Committee (MOHAP/DXB-REC/D.D.D/No.131/2021; MOHAP/DXB-REC/J.J.J./No. 86/2023), Dubai Scientific Research Ethics Committee (DSREC-GL17-2023), and Abu Dhabi Health Research and Technology Ethics Committee (DOH/ZHCD/2023/1316).

## Author contributions

DE, JT, NA, GM, CM, and AS: conceptualization. JT, NA, GM, CM, AS, DE, and The UAE AMR surveillance consortium: Data collection. PN, DE, and JT: formal analysis. PN, AS, JT, NA, GM, CM, and DE: data interpretation. DE and JT: manuscript preparation. DE, JT, NA, PN, GM, CM, AS, FJ, and SW: manuscript review and editing. All named authors have read and agreed to the published version of the manuscript.

## Group members of the UAE AMR Surveillance Consortium

^1^Abiola Senok, College of Medicine, Mohammed Bin Rashid University of Medicine and Health Sciences, Dubai; ^2^Adnan Alatoom, Sheikh Shakhbout Medical City (SSMC), Abu Dhabi; ^3^Agnes-Sonnevend-Pal, University of Pécs, Pécs, Hungary; ^4^Ahmed Abdulkareem Al Hammadi, Tawam Hospital, Al Ain; ^5^Ahmed Elhag Ahmed, UAE University, College of Medicine and Health Sciences, Al Ain; ^6^Ahmed F. Yousef, Department of Biology, Center for Membranes and Advanced Water Technology, Khalifa University, Abu Dhabi; ^7^Alaa MM Enshasy, Dubai Health Authority, Dubai; ^8^Amal Mubarak Madhi, Abu Dhabi Public Health Center, Abu Dhabi; ^9^Amna AlBlooshi, Purelab, Al Ain; ^10^Andreas Podbielski, University Hospital Rostock, Rostock, Germany; ^11^Anju Nabi, Dubai Academic Health Corporation (DAHC), Dubai; ^12^Anup Shashikant Poddar, Al Sharq Hospital, Fujairah; ^13^Arun Kumar Jha, Danat Al Emarat Hospital, Abu Dhabi; ^14^Ayesha Abdulla Al Marzooqi, Abu Dhabi Public Health Center, Abu Dhabi; ^15^Bashir Aden, Khalifa University, Abu Dhabi; ^16^Carole Ayoub Moubareck, College of Natural and Health Sciences, Zayed University, Dubai; ^17^Dean Everett, Department of Pathology and Infectious Diseases, College of Medicine, Khalifa University, Abu Dhabi; ^18^Deeba Jafri, Purelab, Sheikh Khalifa Medical City, Ajman; ^19^Duckjin Hong, Sheikh Khalifa Specialty Hospital (SKSH) RAK; ^20^Emmanuel Fru Nsutebu, Sheikh Shakhbout Medical City, Abu Dhabi; ^21^Farah Ibrahim Al-Marzooq, United Arab Emirates University, Al Ain; ^22^Fatima Al Dhaheri, United Arab Emirates University, Al Ain; ^23^Fouzia Jabeen, Purelab, Sheikh Khalifa Hospital, Abu Dhabi; ^24^Francis Amirtharaj Selvaraj, Sheikh Khalifa Medical City (SKMC), Abu Dhabi; ^25^Ghada Abdel Wahab, Abu Dhabi Agriculture and Food Safety Authority, Abu Dhabi; ^26^Ghalia Abdul Khader Khoder, University of Sharjah, Sharjah; ^27^Gitanjali Avishkar Patil, NMC Specialty Hospital, Abu Dhabi; ^28^Godfred A. Menezes, Department of Medical Microbiology and Immunology, RAK Medical and Health Sciences University, Ras Al Khaimah; ^29^Hadayatullah Ghulam Muhammad, Emirates International Hospital, Al Ain; ^30^Hafiz Ahmad, RAK Hospital, Ras Al Khaimah; ^31^Hala Ahmed Fouad Ismail, PureLab, Al Qassimi Hospital, Sharjah; ^32^Hazim Khalifa, Department of Veterinary Medicine, UAE University, Al Ain; ^33^Husein Alzabi, Sheikh Khalifa General Hospital, Um al Quwain; ^34^Ibrahim Alsayed Mustafa Alhashami, Purelab, Al Qassimi Hospital, Sharjah; ^35^Imene Lazreg, University of Sharjah, Sharjah; ^36^Irfaan Akthar, Mediclinic City Hospital, Dubai; ^37^Jens Thomsen, Abu Dhabi Public Health Center, Abu Dhabi; ^38^John Stelling, WHONET, Boston, United States; ^39^Kaltham Ali Kayaf, Ministry of Climate Change and Environment (MOCCAE), Dubai; ^40^Kavita Diddi, Prime Hospital, Dubai; ^41^Krishnaprasad Ramabhadran, Burjeel Hospital, Abu Dhabi; ^42^Laila Al Dabal, Dubai Academic Health Corporation (DAHC, Dubai); ^43^Laura Thomsen, University of Freiburg, Germany; ^44^Leili Chamani-Tabriz, Clemenceau Medical Center, Dubai; ^45^Madikay Senghore, Khalifa University, Abu Dhabi; ^46^Manal Abdel Fattah Ahmed, PureLab, Ras Al Khaimah; ^47^Maya Habous, Rashid Hospital, Dubai Academic Health Corporation, Dubai; ^48^Moeena Zain, American Hospital Dubai; ^49^Mohamud M. Sheek-Hussein, United Arab Emirates University, Al Ain; ^50^Monika Maheshwari, Al Zahra Hospital, Dubai; ^51^Monika Maheshwari, Medeor 24×7 Hospital, Dubai; ^52^Mubarak Saif Alfaresi, Zayed Military Hospital, Abu Dhabi; ^53^Mushtaq Khan, United Arab Emirates University, Al Ain; ^54^Najiba Abdulrazzaq, Al Kuwait Hospital, Emirates Health Services Establishment, Dubai; ^55^Nehad Nabeel Al Shirawi, Al Fujairah Hospital; ^56^Nesrin Helmy, Mediclinic Al Noor Hospital–Khalifa Street, Abu Dhabi; ^57^Pamela Fares Murad, Abu Dhabi Public Health Center (ADPHC), Abu Dhabi; ^58^Pascal Frey, Berne University Hospital, Berne, Switzerland; ^59^Peter Nyasulu, Department of Global Health, Faculty of Medicine and Health Sciences, Stellenbosch University, South Africa; ^60^Prashant Nasa, NMC Specialty Hospital Al Nahda, Dubai; ^61^Rajeshwari T. A. Patil, Burjeel Medical City, Abu Dhabi; ^62^Rania El Lababidi, Department of Pharmacy Services, Cleveland Clinic Abu Dhabi; ^63^Ratna A. Kurahatti, NMC Royal Hospital Khalifa City A, Abu Dhabi; ^64^Riyaz Amirali Husain, Dubai Hospital, Dubai Academic Health Corporation, Dubai; ^65^Robert Lodu Serafino Wani Swaka, Sheikh Shakhbout Medical City, Abu Dhabi; ^66^Saeed Hussein, Erada Center for Treatment and Rehabilitation, Dubai; ^67^Sameh Soliman, University of Sharjah, Sharjah; ^68^Savitha Mudalagiriyappa, University Hospital Sharjah, Sharjah; ^69^Seema Oommen, Burjeel Medical City, Abu Dhabi; ^70^Shaikha Ghannam Alkaabi, Abu Dhabi Public Health Center, Abu Dhabi; ^71^Simantini Jog, Fakeeh University Hospital, Dubai; ^72^Simantini Jog, King’s College Hospital London Dubai Hills, Dubai; ^73^Siobhan O‘Sullivan, Khalifa University, Abu Dhabi; ^74^Somansu Basu, NMC Specialty Hospital, Al Ain; ^75^Stefan Weber, Purelab, Abu Dhabi; ^76^Sura Khamees Majeed, Al Gharbia Hospitals–Madinat Zayed Hospital; ^77^Syed Irfan Hussein Rizvi, Mediclinic City Hospital, Dubai; ^78^Tibor Pal, University of Pécs, Pécs, Hungary; ^79^Timothy Anthony Collyns, Tawam Hospital, Al Ain; ^80^Yassir Mohammed Eltahir Ali, Animal Wealth Sector, Abu Dhabi Agriculture and Food Safety Authority, Abu Dhabi; ^81^Yousuf Mustafa Naqvi, Department of Health Abu Dhabi (DoH), Abu Dhabi; ^82^Zahir Osman Babiker, Sheikh Shakhbout Medical City (SSMC), Abu Dhabi; ^83^Zulfa Omar Al Deesi, Latifa Maternity and Pediatric Hospital, Dubai.

## Footnotes

^1^ www.whonet.org
