# Supplementary figures and images for: Epidemiology and antimicrobial resistance of Mycobacterium spp. in the United Arab Emirates: a retrospective analysis of 12 years of national antimicrobial resistance surveillance data
Source: Front Public Health. 2024 Jun 14;12:1244353. doi: 10.3389/fpubh.2024.1244353 (PMC11211529; doi:10.3389/fpubh.2024.1244353)

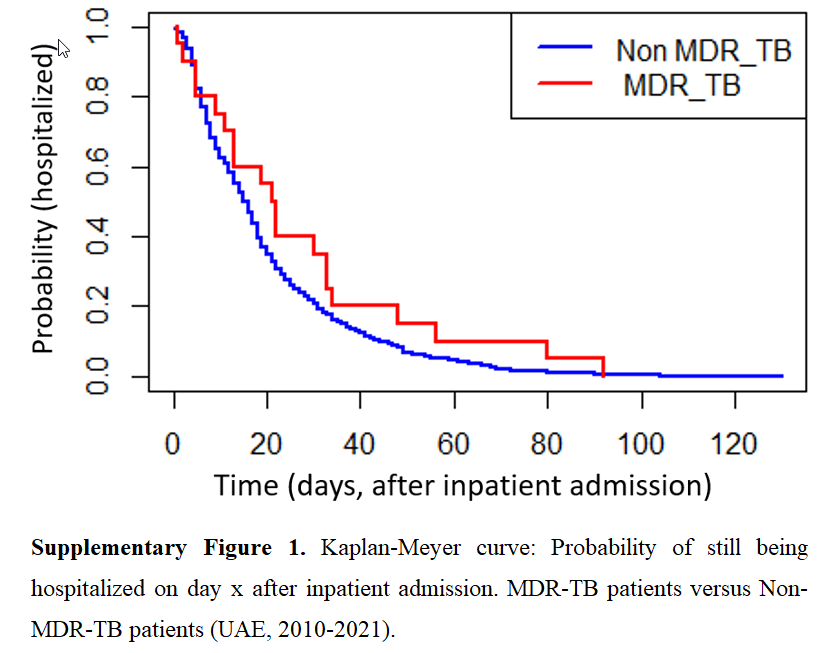

Supplement: Supplementary file 1 [file Image_1.png]
